# Generation of inducible immortalized bone marrow derived cell lines expressing mutant procaspase-1 C284A on a caspase-1 knock-out background

**DOI:** 10.1186/1546-0096-13-S1-O4

**Published:** 2015-09-28

**Authors:** F Kapplusch, F Kulling, S Reinke, M Heymann, S Russ, A Gocht, K Höhne, S Winkler, A Rösen-Wolff, K Anastassiadis, S Hofmann

**Affiliations:** 1 Medizinische Fakultät Carl Gustav Carus, Technische Universität Dresden, Department of Pediatrics, Dresden, Germany; 2 Stem Cell Engineering, Biotechnology Center (BIOTEC), Technische Universität Dresden, Dresden, Germany

## Introduction

Caspase-1, belonging to the family of cystein proteases, is well-known for its involvement in IL-1β and IL-18 maturation. Although, caspase-1 is one of the best described caspase-family-members, open questions concerning autoinflammatory diseases, caused by procaspase-1 variants (e.g. ICE-Fever), remain. ICE-Fever patients suffer from chronic febrile episodes, although procaspase-1 variants show reduced enzymatic activity leading to limited IL-1β secretion. The paradox of reduced IL-1β secretion but increased inflammation led to the hypothesis, that *CASP1*-variants enhance alternative signaling pathways. However, the role of procaspase-1 variants and their interaction partners during IL-1β maturation or NF-κB activation is still poorly understood.

## Objectives

Studies with macrophages and dendritic cells are mainly limited by their low numbers *in vivo* and their difficult maintenance *in vitro*. In order to unravel the pathophysiological mechanisms of caspase-1 related autoinflammation, we aimed to generate inducible immortalized cell lines from transgenic mice expressing an inducible SV40 large T-antigen and mutant procaspase-1 C284A on a caspase-1 and/or receptor-interacting-protein 2 (RIP2) knock-out background.

## Materials and methods

We crossed transgenic mice expressing an inducible SV40 large T-antigen with caspase-1 and RIP2 knock-out mice and generated a murine cell system which is in parallel immortalizable and contains the procaspase-1 C284A variant. After isolation of heterogeneous bone marrow cells, we differentiated cells into macrophages or dendritic cells by the supplementation of different colony stimulation factors and immortalized them by the addition of doxycyclin. Immortalized cells as well as de-induced cells (withdraw of doxycycline) were characterized and used for further stimulation experiments, western blot analysis, microscopy and Co-IPs.

## Results

Inducible immortalized murine caspase-1 and RIP2 knock-out cell lines were generated and stable in long-term culture. Subsequent analysis of corresponding surface markers verified the differentiation status of the generated cell lines. Immortalized cells and de-induced cells were characterized and used for further stimulation experiments, investigating the consequences of enzymatically impaired procaspase-1 variant C284A and their interaction partners. We further established cell lines usable for live cell imaging of procaspase-1 wildtype or variant C284A and its interaction partners RIP2 and ASC.

## Conclusion

In summary, we successfully expanded bone marrow derived macrophages and dendritic cells containing caspase-1 and RIP2 knock-out using conditional immortalization. The generated de-induced cells demonstrate the characteristic immunophenotype of primary cells. They thus represent a physiological model for further studies on the effects of the procaspase-1 variant C284A on inflammatory signaling pathways.

